# Importance of Preconception Reproductive Genetic Screening in Routine Clinical Care

**DOI:** 10.7759/cureus.100572

**Published:** 2026-01-01

**Authors:** Nidhi Basavaraj, Nooruliza Pallathur, Amna G Elbadri Taha, Renu Sharma, Bashir Imam, Aaliya Rahman, Rahma Muse, Sanyam Sharma, Pallavi Shekhawat, Manju Rai

**Affiliations:** 1 Internal Medicine, JSS Medical College, Mysore, IND; 2 Obstetrics and Gynaecology, University of Warwick, Coventry, GBR; 3 Obstetrics and Gynaecology, Al Neelain University, Khartoum, SDN; 4 Internal Medicine, Nepalgunj Medical College, Kathmandu, NPL; 5 Pediatrics, University of Pittsburgh Medical Center Cole, Coudersport, USA; 6 Internal Medicine, Dr. D. Y. Patil Medical College Hospital and Research Center, Pimpri-Chinchwad, IND; 7 Internal Medicine, East Africa University, Bosaso, SOM; 8 Internal Medicine, Government Medical College, Amritsar, IND; 9 Obstetrics and Gynecology, Employees' State Insurance Corporation Model Hospital and Post Graduate Institute of Medical Sciences &amp; Research, Basaidarapur, IND; 10 Biotechnology, Shri Venkateshwara University, Gajraula, IND

**Keywords:** ethical considerations, expanded carrier screening, genetic counseling, health disparities, next-generation sequencing, personalized medicine, polygenic risk scores, preconception genetic screening, reproductive decision-making, reproductive genomics

## Abstract

Preconception reproductive genetic screening (PRGS) is an increasingly important strategy in preventive reproductive healthcare, enabling the identification of carrier status for autosomal recessive and X-linked disorders in asymptomatic individuals prior to conception. Advances in genomic technologies and expanding professional guidelines have shifted screening paradigms from ethnicity-based approaches toward population-neutral expanded carrier screening, underscoring the need for updated clinical and policy perspectives. The objective of this narrative review is to synthesize contemporary evidence on the clinical utility, technological evolution, ethical considerations, and implementation challenges of PRGS in routine care. A comprehensive literature search was conducted across PubMed, Scopus, Cochrane Library, and Web of Science, covering publications from January 2000 to September 2025. Following removal of duplicates and screening of titles, abstracts, and full texts, 80 studies were included in the final narrative synthesis. The reviewed evidence demonstrated that expanded carrier screening using next-generation sequencing improved detection of at-risk couples compared with traditional targeted approaches and supported informed reproductive decision-making. Integration of PRGS with genetic counseling, assisted reproductive technologies, and emerging digital tools such as artificial intelligence-assisted variant interpretation may further enhance scalability and precision. However, significant barriers persist, including variable insurance coverage, limited access to genetic counseling, underrepresentation of diverse populations in genomic databases, and unresolved ethical and psychosocial concerns. Overall, PRGS represents a clinically valuable and ethically complex preventive strategy with significant public health implications. When responsibly implemented with appropriate counseling, equitable access, and robust policy support, PRGS has the potential to reduce the burden of inherited genetic disorders and advance personalized, patient-centered reproductive care.

## Introduction and background

Preconception carrier screening (PCS) and preconception reproductive genetic screening (PRGS) are closely related terms that are often used interchangeably in the literature, although subtle distinctions exist. PCS traditionally refers to the identification of carrier status for autosomal recessive and X-linked disorders in asymptomatic individuals before conception. PRGS is a broader concept that encompasses PCS while emphasizing its integration into reproductive planning, genetic counseling, and public health-oriented preventive strategies, thereby enabling individuals and couples to make informed reproductive decisions aligned with their personal beliefs and values [1]. In this review, the term PRGS is used preferentially to reflect the expanding scope of carrier screening beyond individual risk assessment toward population-level implementation and preventive reproductive healthcare, while PCS is retained when referring specifically to carrier detection practices described in existing guidelines or studies. From a public health perspective, PRGS represents a preventive strategy with the potential to reduce the incidence of severe inherited disorders, decrease long-term healthcare costs, and promote reproductive autonomy through informed decision-making.

Traditionally, PCS focused on a limited number of severe, early-onset genetic disorders characterized by significant morbidity or reduced life expectancy. Early screening programs primarily targeted individuals with a known family history of genetic disease or populations with higher carrier frequencies linked to ethnicity or geographic origin, such as cystic fibrosis in individuals of European ancestry, Tay-Sachs disease in Ashkenazi Jewish populations, and β-thalassemia in Mediterranean regions [2]. While effective in selected groups, this approach relied heavily on family history and ancestry as proxies for genetic risk.

However, most carriers of autosomal recessive and X-linked conditions are clinically asymptomatic and often lack a recognized family history of disease. Consequently, a substantial proportion of affected children are born to parents who are unaware of their carrier status prior to conception. Increasing population admixture and limitations in ethnicity-based risk stratification further reduce the effectiveness of targeted screening models, underscoring the need for more inclusive approaches [3,4].

Advancements in next-generation sequencing (NGS) have addressed these limitations through expanded carrier screening (ECS), a comprehensive, panel-based strategy that enables simultaneous screening for hundreds of genetic conditions irrespective of ethnicity [5]. By improving the detection of carrier status across diverse populations, ECS offers broader and more equitable insights into reproductive risk.

The identification of carrier couples before conception allows consideration of multiple reproductive options, including in vitro fertilization with preimplantation genetic testing, use of donor gametes, adoption, or informed natural conception. Given the complexity and potential psychosocial implications of screening results, genetic counseling remains a cornerstone of PRGS. Pre- and post-test counseling supports accurate interpretation of results and facilitates informed, non-directive reproductive decision-making [6,7].

The primary aim of this narrative review is to define PCS and PRGS, outline their evolution, and highlight the importance of integrating carrier screening into routine clinical practice to prevent inherited genetic disorders and support informed reproductive choices among prospective parents.

## Review

Methodology

This narrative review was conducted to examine the current evidence, clinical practices, technological developments, and ethical considerations surrounding PRGS. A comprehensive literature search was performed using major scientific databases, including PubMed, Scopus, Cochrane Library and Web of Science, covering studies published between January 2000 and September 2025. In addition to peer-reviewed articles, practice guidelines and position statements from leading professional societies such as the American College of Obstetricians and Gynecologists (ACOG), the American College of Medical Genetics and Genomics (ACMG), the European Society of Human Genetics (ESHG), and the National Society of Genetic Counselors (NSGC) were reviewed to ensure that the most current recommendations were incorporated. The search strategy used combinations of keywords and MeSH terms, including “preconception carrier screening,” “expanded carrier screening,” “reproductive genetic screening,” “next-generation sequencing,” “preimplantation genetic testing,” “genetic counseling,” “polygenic risk scores,” “artificial intelligence in genomics,” and “ethical issues in reproductive genetics,” linked using Boolean operators to refine retrieval.

Articles were eligible for inclusion if they focused on preconception carrier screening, expanded carrier screening panels, the integration of next-generation sequencing technologies into reproductive screening, clinical utility and reproductive decision-making, ethical or psychosocial implications of genetic testing, or global and national policy frameworks pertaining to reproductive genetics. Studies were excluded if they were not available in English, lacked a full-text version, duplicated existing data, or examined genetic testing solely for postnatal or adult-onset conditions unrelated to reproductive planning. Reference lists of selected articles were scanned manually to identify additional relevant publications not captured during the primary search.

**Figure 1:**
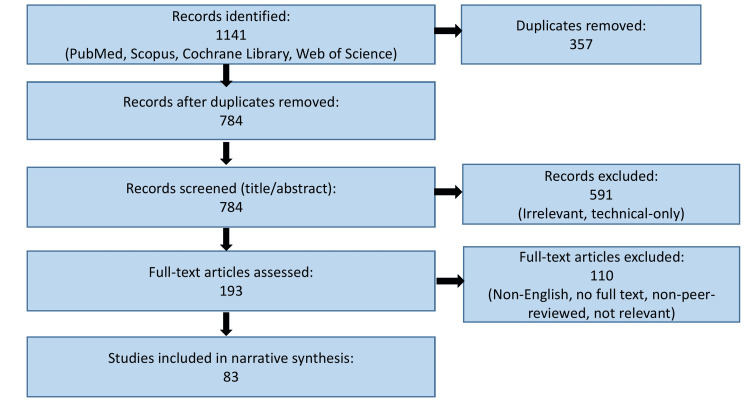
PRISMA-style flow diagram of literature search and study selection PRISMA: Preferred Reporting Items for Systematic Reviews and Meta-Analyses Image credit: Created in BioRender. Sharma S (2025) https://app.biorender.com/illustrations/canvas-beta/69554e71e6f5fdb11744bb61

Given the broad and interdisciplinary nature of PRGS, the findings from the included sources were synthesized narratively. No formal meta-analysis or quantitative assessment was conducted, as the objective of this review was to integrate diverse forms of evidence, scientific, clinical, ethical, and policy-driven, into a cohesive understanding of current practices and emerging directions in preconception genetic screening. This methodological approach allowed for a comprehensive evaluation of PRGS within the context of evolving genomic technologies and global implementation challenges. The literature identification, screening, and inclusion process is illustrated in a Preferred Reporting Items for Systematic Reviews and Meta-Analyses (PRISMA)-style flow diagram (Figure 1).

Current guidelines and global practices

Building on the rationale for population-neutral carrier screening, several professional societies have issued guidance on the clinical implementation of PRGS. The ACOG recommends offering carrier screening ideally before conception to facilitate informed reproductive decision-making [7]. ACOG advises screening for conditions such as cystic fibrosis and spinal muscular atrophy, with reflex testing of the reproductive partner when an individual is identified as a carrier. Although carrier screening does not eliminate genetic risk entirely, it significantly reduces the likelihood of an affected pregnancy and should be documented as a one-time test in the medical record [8].

The ACMG endorses a tiered, population-neutral approach to carrier screening [8]. Rather than detailing each tier in routine clinical practice, ACMG emphasizes broader panels applicable to all individuals, regardless of ethnicity or family history, and discourages reliance on limited, ancestry-based screening strategies in increasingly diverse populations. Expanded screening may be further tailored in the presence of relevant family history or consanguinity, with documentation of both partners’ carrier status recommended to inform future reproductive planning [9].

Similarly, the ESHG supports expanded carrier screening, ideally offered preconceptionally with informed consent, appropriate patient education, and access to comprehensive genetic counseling [10]. Both ACMG and ESHG stress that expanded carrier screening should complement, rather than replace, established prenatal and newborn screening programs, and that ethical principles such as equitable access and reproductive autonomy are central to responsible implementation.

As shown in Table 1, global adoption of preconception carrier screening varies widely across healthcare systems, reflecting differences in national policies, funding structures, and population uptake.

**Table 1:** Global comparison of preconception carrier screening (PCS) policies and practices ACOG: American College of Obstetricians and Gynecologists; ACMG: American College of Medical Genetics and Genomics; NHS: National Health Service; NICE: National Institute for Health and Care Excellence; ECS: Expanded carrier screening.

Country	National Guidelines / Policies	Implementation & Access	Population Uptake
USA	ACOG recommends offering carrier screening to all individuals considering or currently pregnant; ACMG recommends Tier 3 panel (≥1/200 carrier frequency), population-neutral screening [8,9].	Available through private and fertility centers; insurance coverage variable, Medicare doesn’t cover it; increasingly offered in fertility clinics and obstetric practices. Not available in rural areas.	Low to moderate; higher in certain subgroups (fertility patients, Ashkenazi Jewish population); patients from rural areas and with no family history of genetic disorder left behind, there is growing awareness [11,12].
UK	No universal guideline for PCS; NHS offers targeted screening (e.g. thalassemia, sickle cell); NICE recommends risk-based screening [13,14].	Publicly funded for high-risk groups; private expanded screening available; National awareness campaigns are limited.	Low in general population; moderate in high-risk ethnic groups [14,15].
India	No national PCS policy; limited regional screening programs (e.g., thalassemia in certain states); consanguinity-based screening in some regions [16,17].	Mostly limited to urban private sector; minimal public infrastructure; growing private ECS offerings.	Very low overall; higher in communities with consanguineous marriages or known high-risk disorders [17,18].
Israel	National government-funded PCS program; population-wide screening offered for multiple conditions depending on ancestry [19,20].	Fully integrated into the national health system; highly accessible; strong public education programs.	Very high (~90-95%) across eligible populations [18,19].

Effective implementation of guideline-based screening depends not only on testing strategies but also on access to skilled genetic counseling and supportive healthcare infrastructure, particularly in resource-limited settings.

Preconception genetic counseling - coverage, integration & insurance

Genetic counseling is central to translating carrier screening results into informed reproductive decisions, yet its integration into healthcare systems varies widely. In the United States, the insurance coverage for PGC is generally limited to individuals with specific medical indications, such as a family history of genetic disease or belonging to a high-risk ethnic group [21]. Coverage policies vary widely among insurers, and preauthorization is often required [22]. Notably, Medicare currently does not permit genetic counselors to bill independently, creating barriers to access for some patients [23]. However, legislative efforts such as the proposed Access to Genetic Counselor Services Act have sought to address this gap, and independent Medicare billing recognition for genetic counselors remains unchanged as of 2024-2025 [24].

Larger healthcare institutions and academic medical centers often integrate PGC into routine reproductive care through established referral pathways. In contrast, providers in small clinics or rural settings may lack the time, training, or resources to offer or coordinate genetic counseling services effectively [25].

Although billing codes exist for genetic counseling, reimbursement remains inconsistent and frequently inadequate, which further limits its utilization. The inability of genetic counselors to bill Medicare directly results in the underutilization of services among eligible populations [23].

Internationally, countries such as the United Kingdom, Canada, and Israel offer more equitable access to PGC through publicly funded healthcare systems. For example, Israel’s national genetic screening program provides free genetic testing to specific communities based on elevated carrier frequencies and population-specific risks [26].

Beyond counseling access, the choice between targeted and expanded screening approaches further shapes the reach and equity of PRGS programs.

Expanded versus targeted carrier screening panels

Screening strategies in PRGS range from traditional targeted panels to expanded, population-neutral approaches enabled by genomic technologies. Carrier screening aims to identify pathogenic variants associated with autosomal recessive or X-linked disorders in asymptomatic individuals prior to conception, thereby informing reproductive risk and decision-making [21,27].

Historically, carrier screening was offered selectively to individuals from high-risk ethnic groups or those with a relevant family history, an approach referred to as targeted carrier screening [28]. This strategy focused on testing for a limited number of well-characterized mutations prevalent in specific populations and has been a longstanding component of preconception and prenatal care for individuals at increased genetic risk [29]. A well-established example includes screening in individuals of Ashkenazi Jewish descent for conditions such as cystic fibrosis, Gaucher disease, and Tay-Sachs disease [30].

Despite its utility, targeted screening has important limitations, particularly in increasingly admixed populations. It may fail to identify carriers outside recognized high-risk groups or those harboring pathogenic variants not included in restricted panels, leading to missed diagnoses and residual reproductive risk [31].

**Figure 2:**
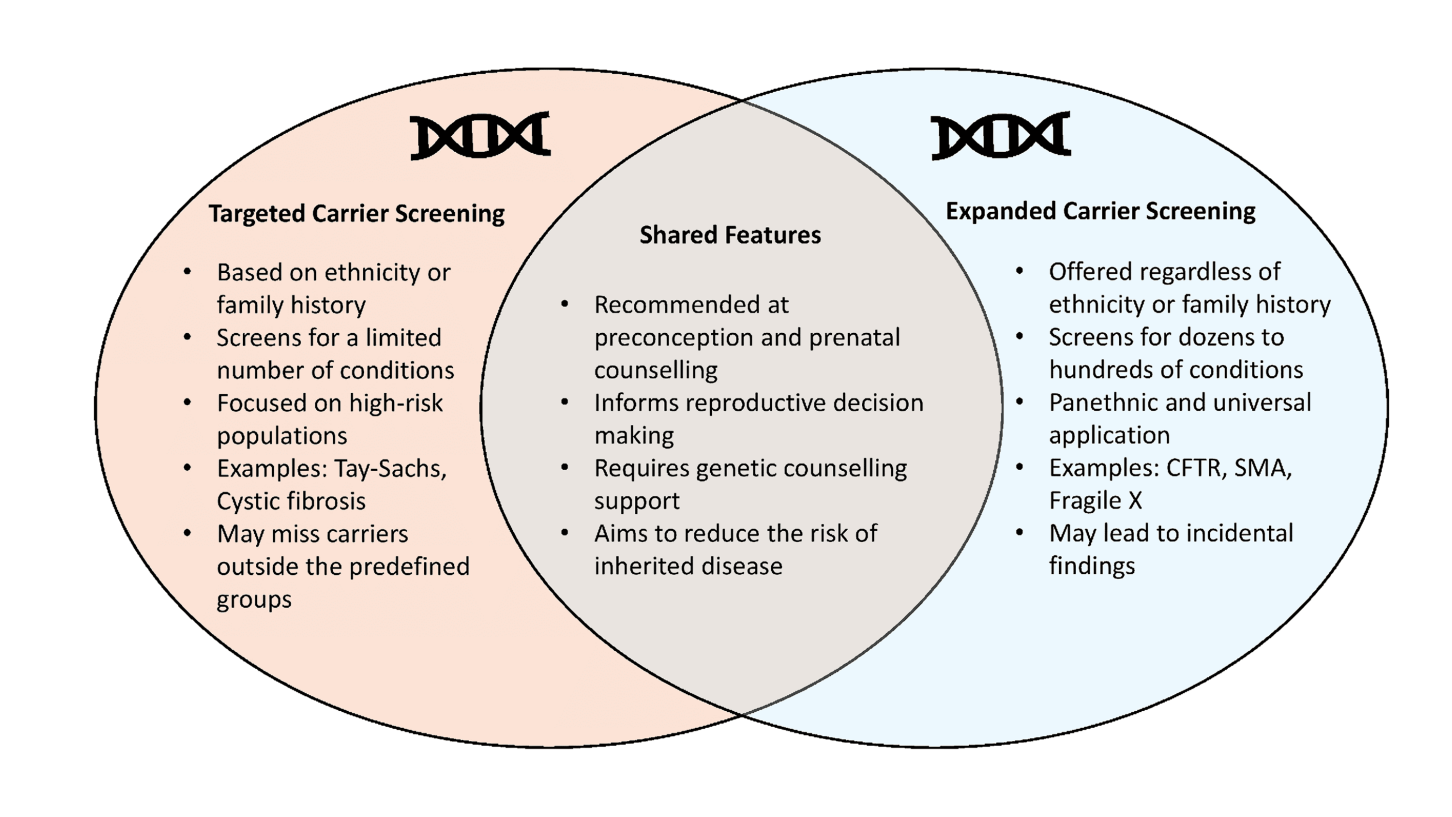
Venn diagram compares the two approaches to carrier screening used in genetic testing Targeted Carrier Screening and Expanded Carrier Screening, along with their shared characteristics. CTFR: Cystic fibrosis; SMA: Spinal muscular atrophy. Image credit: Created in BioRender. Basavaraj N (2025) https://app.biorender.com/illustrations/canvas-beta/69554a4cc2e879b1e6f1e1d5

Advances in NGS have enabled expanded carrier screening (ECS), which allows simultaneous analysis of hundreds of genetic conditions regardless of ancestry [32]. As illustrated in Figure 2, ECS differs from targeted screening in both scope and population applicability while sharing the common goal of identifying carrier status prior to conception.

ECS typically includes conditions such as cystic fibrosis, fragile X syndrome, spinal muscular atrophy, and hemoglobinopathies, and has been shown to substantially improve detection of at-risk couples [33]. Notably, studies suggest that nearly half of cystic fibrosis carriers and a significant proportion of silent spinal muscular atrophy carriers would be missed using traditional targeted panels alone [34].

While ECS offers clear advantages in detection and equity, it also introduces challenges related to result interpretation, patient counseling, and cost [35]. Nevertheless, professional societies including ACOG and ACMG now support the use of expanded, ethnicity-neutral screening panels over targeted approaches when clinically and economically feasible [28,36]. Ongoing advances in sequencing technologies continue to improve the performance, affordability, and clinical integration of ECS, reinforcing its growing role in contemporary PRGS practice.

Next-generation genomic technologies

**Figure 3:**
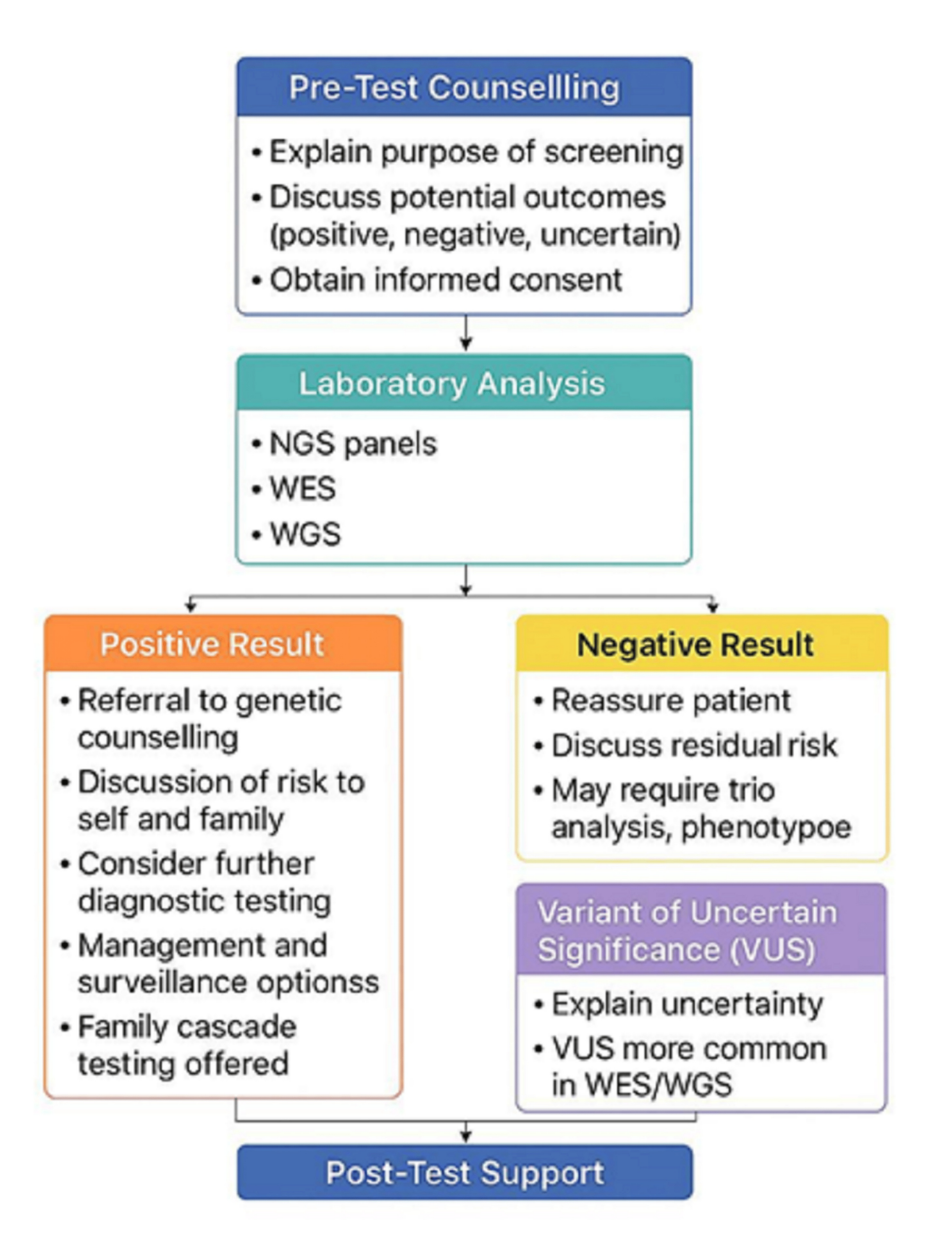
Flowchart illustrating the clinical pathway for genetic screening VUS: Variant of Uncertain Significance; WES: Whole exome sequencing; WGS: Whole genome sequencing. Image credit: Created in BioRender. Pallathur N (2025) https://app.biorender.com/illustrations/canvas-beta/695547d42ce0d3a1577222ef

Next-generation genomic technologies have substantially transformed PCS by enabling comprehensive analysis of parental genomes (Figure 3; Table 2).

**Table 2:** Comparison of NGS, WES and WGS in preconception screening VUS: Variant of uncertain significance.

Feature	NGS (Panel-based)	WES (Whole exome sequencing)	WGS (whole genome sequencing)
Coverage	Selected genes	All protein-coding regions	Entire genome
Diagnostic yield	Moderate	High	Very high
Cost	Most affordable	Moderate	Most expensive
Risk of VUS	Low	Moderate	High
Clinical utility	Good for known inherited disorders	With family history	For unresolved or rare genetic conditions
Used in	Carrier screening panels	Broader diagnostic screening	Comprehensive genomic insight

Approaches such as NGS, whole exome sequencing (WES), and whole genome sequencing (WGS) allow simultaneous assessment of multiple genes, improving identification of carriers for monogenic disorders and supporting informed reproductive decision-making through multidisciplinary clinical collaboration [37].

NGS-based gene panels typically target curated sets of genes associated with common inherited conditions, including cystic fibrosis and spinal muscular atrophy. These panels are relatively cost-effective and clinically accessible, with straightforward interpretation compared to broader sequencing approaches. However, diagnostic yield remains moderate, and multi-gene panels are associated with a substantial burden of variants of uncertain significance (VUS), reported in approximately one-third of cases in large cohort studies [38]. As a result, genetic counseling is essential to support accurate interpretation and mitigate anxiety related to uncertain or incidental findings [37,39].

WES focuses on protein-coding regions, which represent a small proportion of the genome but harbor the majority of known pathogenic variants. In prenatal and pediatric settings, WES has demonstrated diagnostic yields ranging from approximately 28% to 42%, particularly in individuals with structural anomalies [40,41]. Compared with targeted panels, WES increases the likelihood of detecting VUS, with reported rates of around 9% in pediatric cohorts [42]. Interpretation typically requires trio-based sequencing, detailed phenotypic correlation, and multidisciplinary review to improve variant classification and reduce psychosocial burden.

WGS offers the most comprehensive genomic assessment by analyzing both coding and non-coding regions. Although it provides a modestly higher diagnostic yield than WES in pediatric populations, it is associated with higher costs, longer analysis times, and an increased frequency of incidental or ambiguous findings [43]. Strategies such as trio-based analysis and phenotype-driven filtering have been shown to reduce VUS rates compared with standard panel testing, improving clinical interpretability [38].

Across all genomic approaches, interpretation and communication of VUS and incidental findings remain key clinical and ethical challenges. Misclassification of variants can result in false-positive findings, unnecessary interventions, and increased parental distress [41]. Accordingly, genetic counseling must be integrated throughout the screening process to ensure that individuals and couples understand the limitations, probabilistic nature, and potential implications of genomic results, including options for follow-up or non-disclosure.

As genomic data generation continues to expand, digital tools and artificial intelligence are increasingly being used to support variant interpretation, risk assessment, and clinical integration, further shaping the evolving landscape of PRGS.

Artificial intelligence (AI), polygenic risk scores (PRS), and digital platforms

Digital innovations, including artificial intelligence and polygenic risk modeling, are increasingly being explored to enhance the precision and scalability of PRGS.

AI in Variant Interpretation

**Figure 4:**
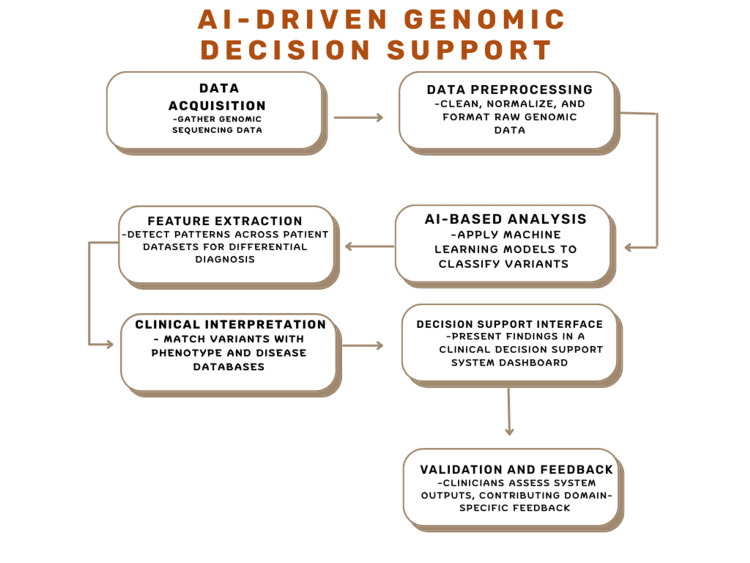
Workflow of an AI-driven genomic decision support system Image credit: Created by Taha AG using Microsoft PowerPoint (Microsoft Corp., Redmond, WA, USA).

AI has become an important enabling tool in healthcare, supporting clinical databases, health information systems, and decision-making frameworks [44,45]. In reproductive medicine, AI has been applied across prenatal screening, non-invasive prenatal testing (NIPT), and preimplantation genetic testing (PGT), including embryo assessment and prediction of implantation potential within assisted reproductive technologies [46] (Figure 4).

In the context of PRGS, AI contributes primarily to improved variant interpretation and risk stratification. While traditional carrier screening relied on monogenic testing and family history [47], newer AI-powered algorithms and multivariate risk models support personalized risk prediction and clinical decision-making [48]. Tools such as VarChat integrate variant pathogenicity data with ACMG criteria, facilitating more efficient and standardized genetic counseling workflows [48].

Emerging studies also suggest a potential role for AI in embryo selection based on projected polygenic disease risk in addition to chromosomal normality; however, these applications remain experimental and are the subject of ongoing ethical and clinical debate [49].

Use of PRS in Reproductive Risk Prediction

Polygenic risk scores or PRS estimate genetic susceptibility to complex traits by aggregating the effects of multiple single nucleotide polymorphisms identified through large genomic datasets [50,51]. In reproductive health, PRS have been explored for risk prediction in conditions such as endometriosis and ovarian cancer, as well as in experimental applications within PGT for polygenic disorders [52-54].

Despite their theoretical promise, the clinical utility of PRS in PRGS remains limited. Predictive accuracy varies across populations, particularly in genetically diverse groups, and current evidence does not support routine use of PRS for embryo selection [51-53]. Ethical concerns and technical limitations have led several experts to caution that PRS-based reproductive decision-making is not yet ready for widespread clinical implementation [52]. Patient responses to PRS information are heterogeneous, with reports of both empowerment and increased anxiety related to probabilistic risk estimates [51].

Digital Tools and Online Screening Interfaces: Benefits and Risks

Digital health technologies are increasingly integrated into PRGS delivery, supporting remote screening, tele-genetic counseling, and standardized care pathways [55-57]. These platforms have the potential to improve accessibility, reduce costs, and facilitate early reproductive risk assessment, particularly in settings with limited specialist availability [56,57].

However, widespread adoption of digital tools introduces important challenges, including data privacy and security risks, algorithmic bias, limited interoperability, and variable clinical validation [58]. While well-designed interfaces may reduce provider burden, poorly integrated systems can contribute to alert fatigue and workflow inefficiencies in clinical practice [59].

Collectively, these digital and genomic innovations offer important opportunities to enhance PRGS but must be implemented cautiously, with appropriate clinical oversight and ethical safeguards to ensure responsible use.

Ethical, legal, and psychosocial considerations

The responsible implementation of PRGS requires careful consideration of individual rights, cultural values, and the broader societal implications of genetic information. Respect for individual autonomy is a foundational principle in reproductive health; however, interpretations of autonomy vary across cultural contexts. In collectivist societies, reproductive decisions may involve extended family members or be shaped by community norms, complicating Western models of informed consent centered solely on individual choice and underscoring the need for culturally adaptive counseling frameworks that support shared decision-making while preserving patient autonomy [60].

In PRGS, informed consent must extend beyond a procedural formality to include clear communication about the scope of testing, the limitations of results, and potential implications for both partners and future offspring [61]. ECS panels often generate large volumes of data with variable clinical relevance, which can overwhelm patients and hinder meaningful comprehension if not adequately supported through counseling [61].

Genetic counseling is therefore central to ethical PRGS implementation and should follow a nondirective approach, providing unbiased information and psychosocial support without steering reproductive choices [62]. Nonetheless, subtle directive influences may arise, particularly in sociocultural settings where disability-related stigma or social pressure shapes decision-making. Ongoing evaluation of counseling practices is essential, especially within population-level screening initiatives, to minimize latent bias and safeguard reproductive autonomy [62].

Psychological responses to carrier or at-risk status are heterogeneous and influenced by individual, relational, and cultural factors. Common reactions include anxiety, guilt, uncertainty, and stigmatization [63]. While genetic counseling can reduce decisional conflict and improve understanding, ambiguous findings-particularly variants of uncertain significance-may increase distress and confusion, and can contribute to interpersonal strain when partners perceive unequal reproductive risk [63,64]. These challenges are further amplified in populations with limited health literacy or limited access to genetic services, emphasizing the importance of integrated psychosocial support within PRGS programs [64].

Given the sensitivity of genetic data, robust legal protections are critical to prevent discrimination and misuse. Legal frameworks vary widely across jurisdictions. In the United States, the Genetic Information Nondiscrimination Act (GINA) protects against genetic discrimination in health insurance and employment but does not extend to life, disability, or long-term care insurance [65]. In contrast, countries such as the United Kingdom and Germany provide broader protections, while many low- and middle-income countries lack comprehensive legislation, leaving individuals vulnerable to privacy violations and discrimination [65].

Social stigma remains a significant concern, particularly for female carriers, who may experience reduced marriage prospects, internalized blame, or social exclusion in settings where fertility and childbearing are closely tied to social identity [66]. Consequently, PRGS programs must be designed to avoid reinforcing existing gender or socioeconomic inequities and to promote equitable, culturally sensitive access to genetic services [66].

Addressing these ethical, legal, and psychosocial considerations is essential to ensure that PRGS translates into meaningful clinical benefit and truly informed reproductive decision-making.

Clinical utility and impact on reproductive decision-making

PCS has substantial clinical utility by enabling early identification of carrier status for autosomal recessive and X-linked disorders, thereby supporting informed reproductive planning before pregnancy is initiated. By identifying at-risk couples preconceptionally, PCS facilitates proactive decision-making aligned with individual values and risk tolerance.

The clinical impact of screening is underscored by large population-based studies. In an analysis of over 300,000 individuals undergoing ECS, Haque et al. reported that nearly 24% were carriers of at least one condition, and approximately one in 175 couples were identified as being at risk for transmitting a serious genetic disorder, many of whom would not have been detected using traditional ethnicity-based panels [67]. These findings highlight the added value of ECS in identifying reproductive risk beyond conventional approaches.

**Figure 5:**
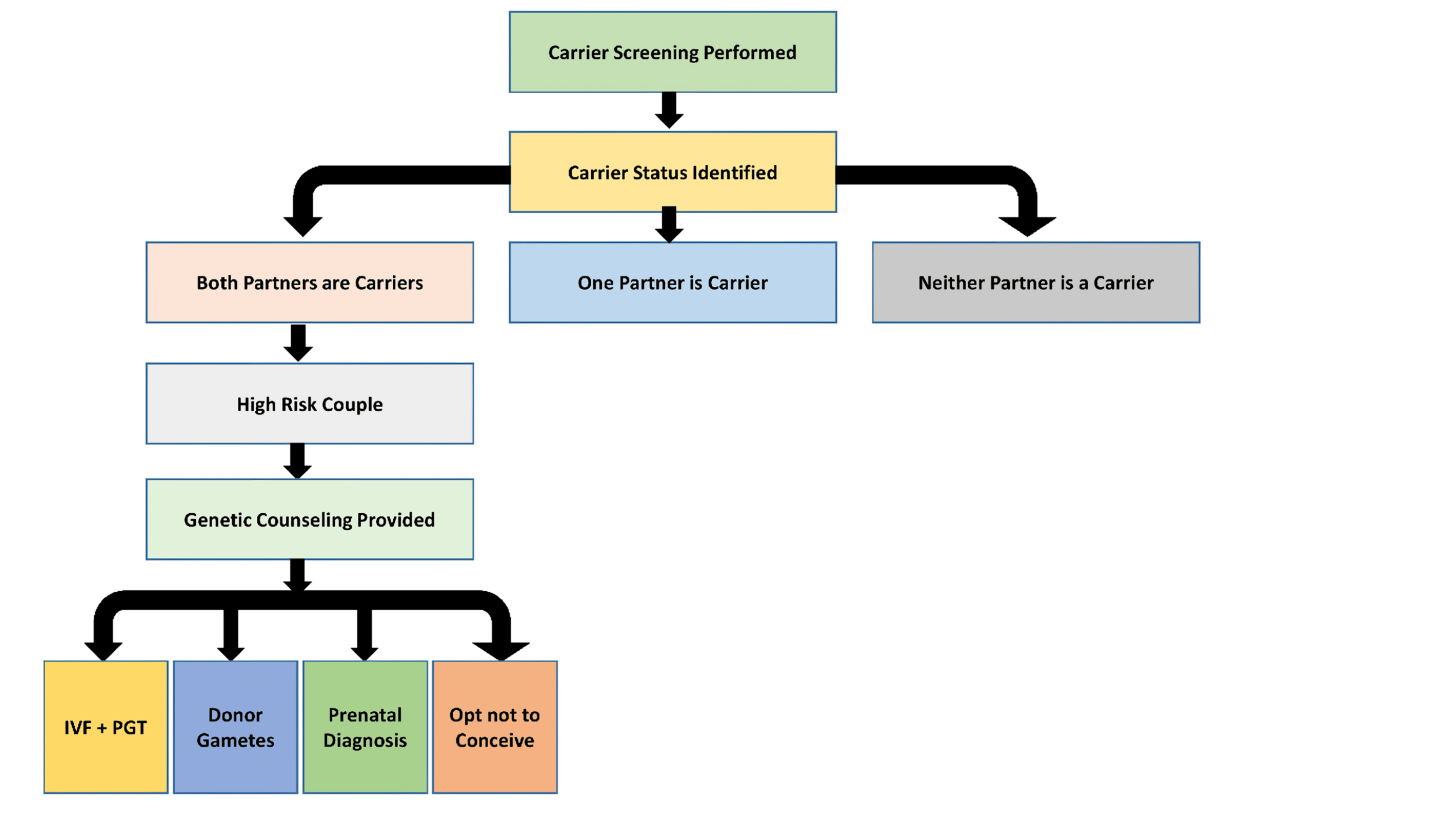
Reproductive decision-making pathway following carrier screening IVF: In vitro fertilization; PGT: preimplantation genetic testing. Image credit: Created in BioRender. Taha AG (2025) https://app.biorender.com/illustrations/canvas-beta/69554d162ce0d3a15776a8ec

Identification of carrier status prior to conception can influence downstream reproductive behavior. As illustrated in Figure 5, screening results may inform a range of reproductive pathways, from further diagnostic testing to modification of conception strategies.

In a prospective cohort study, Ghiossi et al. found that 77% of at-risk couples reported making at least one reproductive change following the ECS results, including pursuit of preimplantation genetic testing, prenatal testing, or reconsideration of natural conception [68].

PCS also plays an important role in assisted reproduction. In fertility clinics, carrier screening is increasingly incorporated into pre-in vitro fertilization (IVF) evaluations, and when combined with PGT, it can substantially reduce the likelihood of transmitting severe monogenic disorders [69]. For couples with known carrier status, this approach may also mitigate the need for complex decision-making following prenatal diagnosis.

Nevertheless, the impact of PCS is shaped by contextual factors such as cultural background, socioeconomic status, and access to reproductive technologies. Some couples may elect not to alter reproductive plans due to personal beliefs, financial constraints, or limited access to genetic counseling and assisted reproductive services [70]. Psychosocial responses to screening vary, with some individuals experiencing reassurance and others reporting anxiety or decisional conflict, particularly in the setting of uncertain results. These considerations underscore the importance of comprehensive, non-directive genetic counseling to support informed interpretation and decision-making [71].

Overall, while the clinical benefits of PCS and ECS are well established, their translation into meaningful reproductive outcomes depends on equitable access, effective counseling, and integration within diverse healthcare settings.

Implementation challenges and health disparities

Despite the growing clinical utility of PRGS, its integration into routine care faces significant implementation challenges, particularly related to access, infrastructure, health literacy, and representation in genomic data. These barriers are compounded in low-resource settings, where disparities are most pronounced.

One of the most persistent challenges is cost-related inaccessibility. In many countries, PRGS and counseling are not universally covered by health insurance, making them financially inaccessible to low- and middle-income individuals. In the United States, for instance, Medicare does not permit genetic counselors to bill directly, and private insurance reimbursement varies widely, leading to inconsistent coverage [72]. Out-of-pocket expenses for ECS and follow-up procedures such as PGT can be prohibitively expensive, limiting uptake among socioeconomically disadvantaged groups [68].

Infrastructural limitations further hinder equitable access. Many rural or underserved communities lack specialized services or trained genetic professionals. Primary care providers may also lack the time, training, or referral pathways to offer genetic counseling as part of routine reproductive care [73]. Additionally, low genetic literacy among both providers and patients is a well-documented barrier. Misconceptions about genetic risk, fear of stigmatization, and cultural resistance to genetic testing can prevent individuals from seeking screening, even when available [74]. Educational interventions and culturally sensitive counseling frameworks are essential to address these gaps.

Another major concern is the underrepresentation of non-European populations in genomic research, which undermines the accuracy and equity of genetic risk assessment. More than 80% of genome-wide association study (GWAS) participants to date have been of European ancestry, despite global population diversity [75]. This skew limits the predictive value of genetic tests in underrepresented populations and contributes to misclassification of variants, ultimately reinforcing health disparities. Expanding genomic datasets to include diverse ancestral groups is imperative for improving the accuracy and generalizability of PRGS results [76].

These challenges are especially acute in low- and middle-income countries, where health systems are often overwhelmed by competing public health priorities. For example, in Cameroon, a lower-middle-income country, limited funding, healthcare infrastructure, and trained personnel have restricted access to genetic services, which remain concentrated in a few urban centers [77]. Moreover, communicable disease burdens often overshadow investment in preventive genetic care. Nevertheless, targeted pilot programs and public-private partnerships in countries such as India and South Africa have shown promise in increasing awareness and access to preconception screening, particularly in high-risk communities [78].

These limitations highlight the need for innovative, scalable, and equity-focused strategies to guide the future of PRGS.

Emerging research and future directions

The landscape of PRGS is rapidly evolving toward broader, more integrated, and personalized approaches. One major shift involves proposals for universal or population-wide carrier screening, moving beyond selective, risk-based models. Pilot programs in countries like the Netherlands and Australia have demonstrated the feasibility and acceptability of "screen-all" models, with high uptake and favorable patient attitudes when implemented with appropriate education and counseling infrastructure [79,80]. These studies emphasize that universal screening can identify at-risk couples who would have been missed by traditional ancestry- or family-history-based strategies.

Simultaneously, there is increasing emphasis on integrating genetic data into electronic health records (EHRs), paired with AI-driven reproductive risk dashboards. These tools aggregate genomic, phenotypic, and family history data to deliver real-time, personalized risk assessments at the point of care [81]. By leveraging clinical decision support systems, such platforms can prompt timely referrals to genetic counseling, suggest tailored testing panels, and track reproductive outcomes [82]. As interoperability standards improve, such integrations promise to enhance screening efficiency, reduce manual workload, and improve the continuity of care.

Moreover, future models of reproductive screening are likely to incorporate multi-omics technologies, including epigenomics, transcriptomics, and metabolomics, enabling deeper insights into reproductive risk beyond single-gene mutations [83]. When combined with genomic data, these approaches could allow for precision reproductive care tailored to individual biology. For example, integrating maternal microbiome and epigenetic profiles may one day predict not only genetic risks but also modifiable environmental factors affecting fetal development.

Together, these emerging directions signal a paradigm shift toward more proactive, predictive, and personalized reproductive health care. However, their success will depend on rigorous validation, ethical oversight, equitable access, and continued patient education to avoid widening existing disparities.

## Conclusions

PRGS represents a powerful preventive strategy in reproductive healthcare, enabling early identification of inherited risk and supporting informed decision-making before conception. The shift from targeted, ancestry-based screening to expanded, population-neutral approaches-enabled by advances in NGS and digital decision-support tools-reflects the growing precision of reproductive medicine.

Despite well-established clinical utility, widespread implementation remains constrained by cost barriers, uneven access to genetic counseling, infrastructure limitations, and underrepresentation of diverse populations in genomic databases. Ethical, legal, and psychosocial considerations further highlight the importance of culturally sensitive, non-directive counseling and robust policy protections.

From a clinical and policy perspective, the routine offering of preconception expanded carrier screening, integrated with accessible genetic counseling and supported by equitable reimbursement frameworks, should be prioritized to maximize public health impact while safeguarding reproductive autonomy. When responsibly implemented, PRGS has the potential to substantially reduce the burden of inherited genetic disorders and advance personalized, patient-centered reproductive care.
